# Global burden and trends of self-harm from 1990 to 2021, with predictions to 2050

**DOI:** 10.3389/fpubh.2025.1571579

**Published:** 2025-05-14

**Authors:** Li Xie, Liangchen Tang, Yixin Liu, Zhenchao Dong, Xiaojun Zhang

**Affiliations:** Emergency Department, The People's Hospital of Danyang, Affiliated Danyang Hospital of Nantong University, Danyang, China

**Keywords:** self-harm, disease burden, mortality, DALYs, global

## Abstract

**Background:**

Self-harm has become a major public health problem globally. Data on the burden of self-harm in this study were taken from the GBD 2021. This study aimed to quantify historical trends (1990–2021) in the global burden of self-harm across genders, age groups, and regions, and project future changes (2022–2050) through Bayesian forecasting models.

**Methods:**

Based on the seven GBD super-regions, the burden of self-harm was analyzed by region, age, and gender from 1990 to 2021. Hierarchical statistical approach was used to predict trends in global and regional changes in the burden of self-harm, 2022-2050.

**Result:**

In 2021, the global DALYs and death counts from self-harm were 33.5 million (95% UI: 31.3-35.8) and 746.4 thousand (95% UI: 691.8-799.8). The region with the highest number of DALYs and deaths is South Asia and the highest age-standardized rates of DALYs and mortality were in central Europe, eastern Europe, and central Asia. Globally, the burden of self-harm was higher for males than for females. DALYs rates were highest among adolescents and young adults (20-29 years), whereas mortality rates showed a predominantly age-progressive pattern with the highest burden observed in middle-aged and older populations, albeit with a modest decline in the oldest age groups. Forecasting models showed a sustained decline in the global burden of self-harm from 2022-2050.

**Conclusion:**

The results highlight the need for policymakers to allocate resources to high-burden regions (e.g., South Asia and Eastern Europe), to implement gender- and age-specific prevention programs, and to strengthen cross-sectoral collaboration to address the underlying social determinants of self-harm. The findings call for strengthened mental health services and targeted interventions to effectively respond to and reduce the devastating impact of self-harm on individuals and the global community.

## Introduction

Self-harm, defined as the intentional infliction of direct injury to body tissues, has become an important public health problem worldwide (1, 2). Although the etiology of self-harm is complex and includes psychological, social, and cultural factors, the behavior is prevalent across populations and age groups and has serious implications for individual health (3–5). According to the Global Burden of Disease Study 2019, self-harm contributes to a significant number of deaths globally, up to 121,216. In addition, self-harm results in 4,142,557 disability-adjusted life years (DALYs) (6). The burden of self-harm is unevenly distributed, with regions such as Europe and high-income countries having particularly high rates of suicide and self-harming behaviors (7, 8).

Previous studies have focused on specific populations or regions, often using different methods and definitions (9–11). For example, some studies have relied on hospital-based data, which may not capture self-harm behaviors that do not result in hospital visits, leading to potential underestimation of prevalence (12–14). Others have used community-based surveys with varying definitions of self-harm, making cross-study comparisons difficult (13, 15, 16). These studies have provided valuable insights into the risk factors, prevalence, and psychological basis of self-harm. However, these studies are often limited by small sample sizes, cross-sectional designs, and a lack of comprehensive comparable data across countries and regions. These systematic gaps in methodology and scope underscore the necessity for harmonized global surveillance systems. Additionally, the heterogeneity in study methods, such as retrospective self-reporting versus real-time data collection, raises questions about data reliability and validity. Furthermore, most studies focus on single time points and fail to consider temporal trends or the evolution of self-harm behaviors over time. This is a significant limitation because self-harm behaviors are influenced by dynamic factors, such as cultural shifts, technological changes (e.g., the role of social media), and evolving public health policies. To address both the spatial incomparability and temporal limitations, the latest GBD 2021 advances address these limitations through three pivotal innovations (17): (1) explicit incorporation of the COVID-19 pandemic’s direct and indirect impacts on disease burden; (2) integration of 19,189 novel data sources, significantly improving precision in characterizing self-harm epidemiology (particularly non-hospitalized cases); and (3) implementation of a mutually exclusive and collective exhaustive cause hierarchy model that rigorously disentangles interactions between self-harm and comorbid disease burdens—an analytical advancement absent in prior GBD studies. Few studies have conducted long-term projections or integrated multilevel data across regions to provide a global perspective, which is critical for public health planning, particularly in guiding (1) early warning systems for emerging risk clusters, (2) age-specific prevention strategies, and (3) cross-sectoral resource allocation—as emphasized in WHO National suicide prevention strategies.

While recent studies, such as Hu et al. (18), have provided critical insights into mental health and self-harm burdens in China using GBD 2021 data, our study expands this work through a global analysis of self-harm trends across 204 countries, projections to 2050, and sociodemographic drivers (e.g., male-dominated mortality patterns). Unlike Hu et al.’s China-focused integration of self-harm with mental disorders, we isolate self-harm as a distinct public health challenge, identifying scalable interventions (e.g., pesticide regulation) for high-burden regions. These distinctions highlight our unique contribution to addressing geographic disparities in prevention strategies.

The Global Burden of Disease (GBD) study provides a comprehensive and systematic approach to understanding the burden of self-harm on a global scale (19). Using a wide range of data sources and advanced modeling techniques, the GBD provides consistent and comparable estimates of mortality and morbidity associated with self-harm (20). This includes detailed disaggregation by age, sex, and geographic location, as well as trends over time. This study aims to systematically analyze the global, regional, and national burden of self-harm from 1990 to 2021 and to project the burden to 2050, based on data from the Global Burden of Disease Study 2021 (GBD 2021), thus providing valuable insights for addressing future public health challenges (17).

## Methods

### Data sources

The GBD 2021 estimates the incidence, prevalence, mortality, and DALYs of 371 diseases and injuries for 204 countries and territories using a harmonized model based on data from multiple sources, including global censuses, disease registries, cause-of-death registries, and risk factor surveillance. The disease burden of estimation model used for GBD has been described in detail in previous studies (20). The data on self-harm in this study were taken from the GBD 2021, and the selected indicators include counts and rates of DALYs and self-inflicted deaths and their 95% uncertainty intervals (UI) for self-harm, by region, sex, and age, globally, 1990–2021. Age-standardized rates are estimated using the GBD world standard population as a reference (21). According to the International Statistical Classification of Diseases and Related Health Problems, 9th (ICD-9) and 10th (ICD-10) Revisions, the codes for self-harm are E950-E959 for ICD-9 and X60-X64.9, X66-X83.9, and Y87.0 for ICD-10. The GBD 2021 was approved by the University of Washington Institutional Review Board. This study was not approved by the University of Washington because the GBD 2021 data input source tool and visualization tool are open access for use. We downloaded country-level DALYs and death data from the GHDx website in csv format (accessed on July 9, 2024). Detailed data sources are available on the GBD 2021 data input source tool.^1^

### Geographical locations reported

This study estimated the burden of self-harm in 204 countries and territories worldwide, which the GBD categorized according to epidemiological patterns into seven super-regions, central Europe, eastern Europe, and central Asia, high income, Latin America and Caribbean, North Africa and Middle East, South Asia, Southeast Asia, east Asia, and Oceania, and Sub − Saharan Africa. In addition, the super-regions are further divided into 21 regions based on geographic and epidemiological similarities (Supplementary Table S1).

### Forecasting

The GBD 2021 used nested Bayesian meta-regression models to predict the self-harm burden. Based on age-, sex-, year-, and region-specific mortality and DALYs rates for self-harm, the corresponding burden of self-harm was projected to 2050 using the projected population. Predictions were conducted using factors that are key drivers of health, including socio-demographic index (SDI) and risk factor exposures captured by the GBD 2021. SDI is a composite of the total fertility rate for women under 25 years of age, the average educational attainment of the population aged 15 years and over, and per capita income, and is derived from the geometric mean of these three values. The closer the SDI is to 0, the lower the population’s health and development potential, and the closer the SDI is to 1, the higher it is. SDI reflects regional health levels and socioeconomic disparities and is an important factor in moderating health inequalities between regions. For mortality, a mixed-effects model with SDI and time as the main covariates modeled the burden of self-harm. The combined effects of causal risk factors were incorporated into the model as adjustment offsets. To account for residual variability not explained by the covariates, autoregressive integrated moving average (ARIMA) models with drift reduction were employed. It should be clarified that the ARIMA model was derived from internal GBD and no separate ARIMA model was written for this study (17). Mortality projections use a cascading mortality model to restrict cause-specific projections to successively deeper levels of the GBD cause hierarchy, thereby ensuring robust estimates of cause-specific mortality. For nonfatal causes, disease prevalence was estimated through SDI-driven mixed-effects modeling. By combining these prevalence estimates with cause-specific disability weights from the GBD 2019, years lived with disability (YLD) were calculated. The YLD was then aggregated with the years of life lost (YLL) to derive DALYs. The quantification of the estimates is all based on 500 samples processed through a multi-stage computational pipeline, with 95%UI defined by the 2.5th percentile and 97.5th percentile of the distribution of results (17). The detailed methodology has been described by the GBD 2021 Forecasting Collaborators project team in previous studies (17).

For COVID-19, the projection model for the GBD takes into account the indirect effects of the pandemic, including the effects of vaccine coverage, schooling (as measured by educational attainment), and the economy (as measured by per capita income) (17). Given the severe data limitations and reporting lags after 2021, and the fact that there is still considerable uncertainty about the potential long-term direct and indirect impacts of COVID-19 mortality, the model assumes that the number of COVID-19 deaths and DALYs will decline linearly to zero between 2023 and 2030 (17). It is important to be clear that this assumption is speculative, and it is undeniable that such models still struggle to unambiguously predict changes in the burden of self-harm (22).

**Figure 1:**
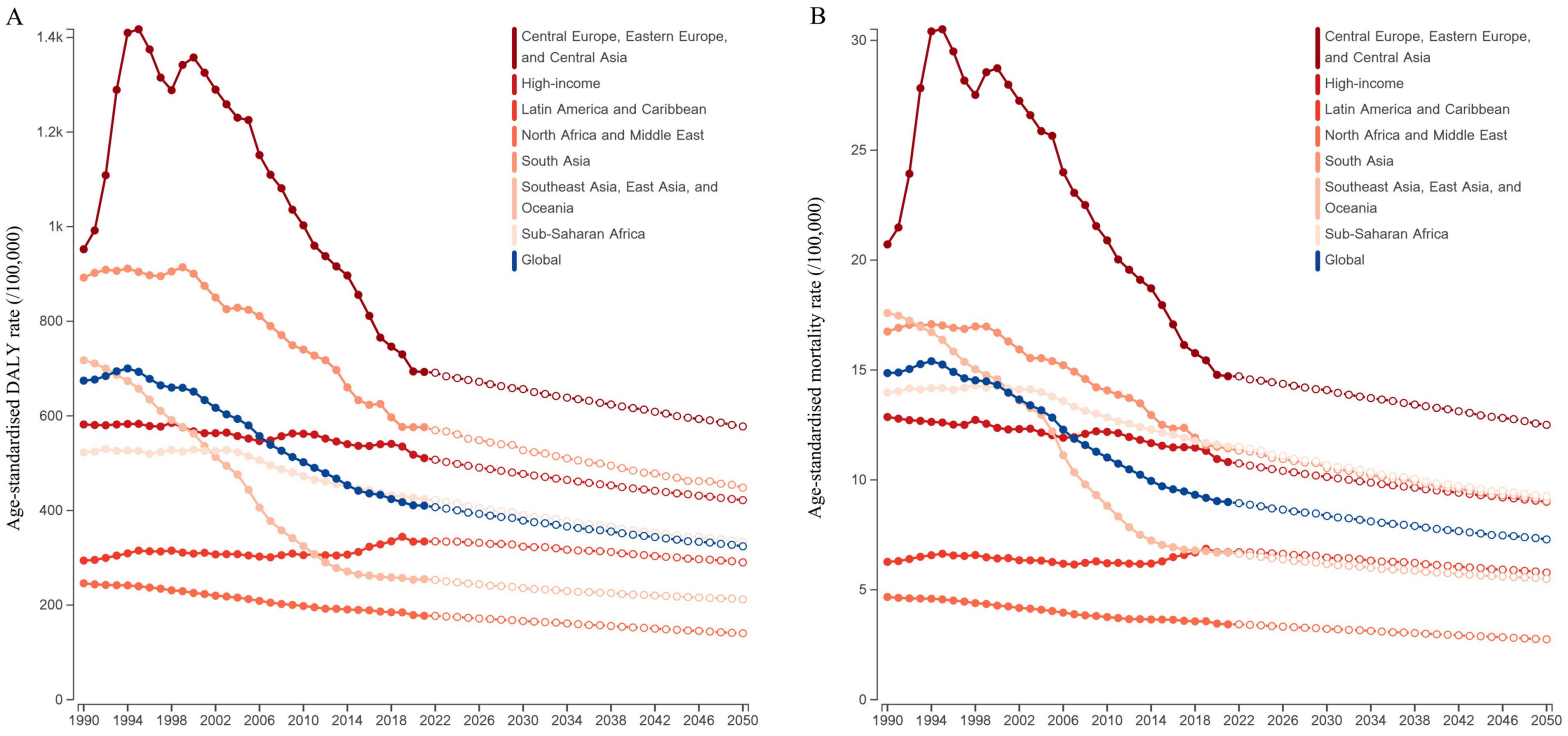
The age distribution of DALYs and mortality rates for self-harm by GBD super-region in 2021. **(A)** DALYs rate for self-harm. **(B)** Mortality rate for self-harm.

**Figure 2:**
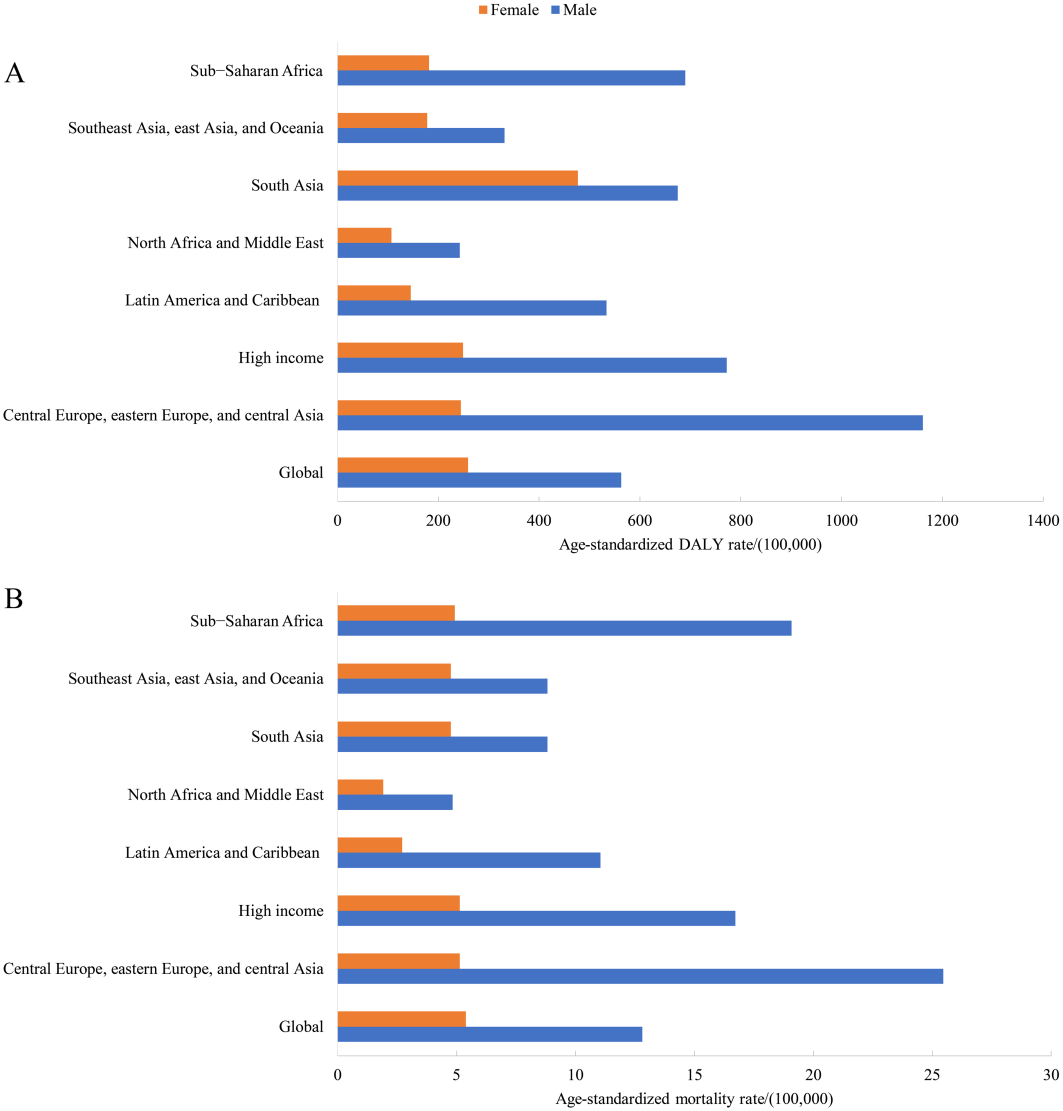
The gender distribution of age-standardized DALYs and mortality rates for self-harm by GBD super-region in 2021. **(A)** Age-standardized DALYs rates for self-harm. **(B)** Age-standardized mortality rate for self-harm.

All the data used in this study were obtained from the estimated data in the GBD 2021 data input source tool, where maps and projections were obtained from the GBD visualization tool. In addition, Excel 2019 was used solely for formatting bar and line charts (Figures 1, 2), and that no statistical modeling, forecasting, or uncertainty intervals calculations were conducted. The analysis used estimated data downloaded from GBD 2021 without combining age groups and districts.

## Results

### The global disease burden of self-harm in different regions

**Figure 3:**
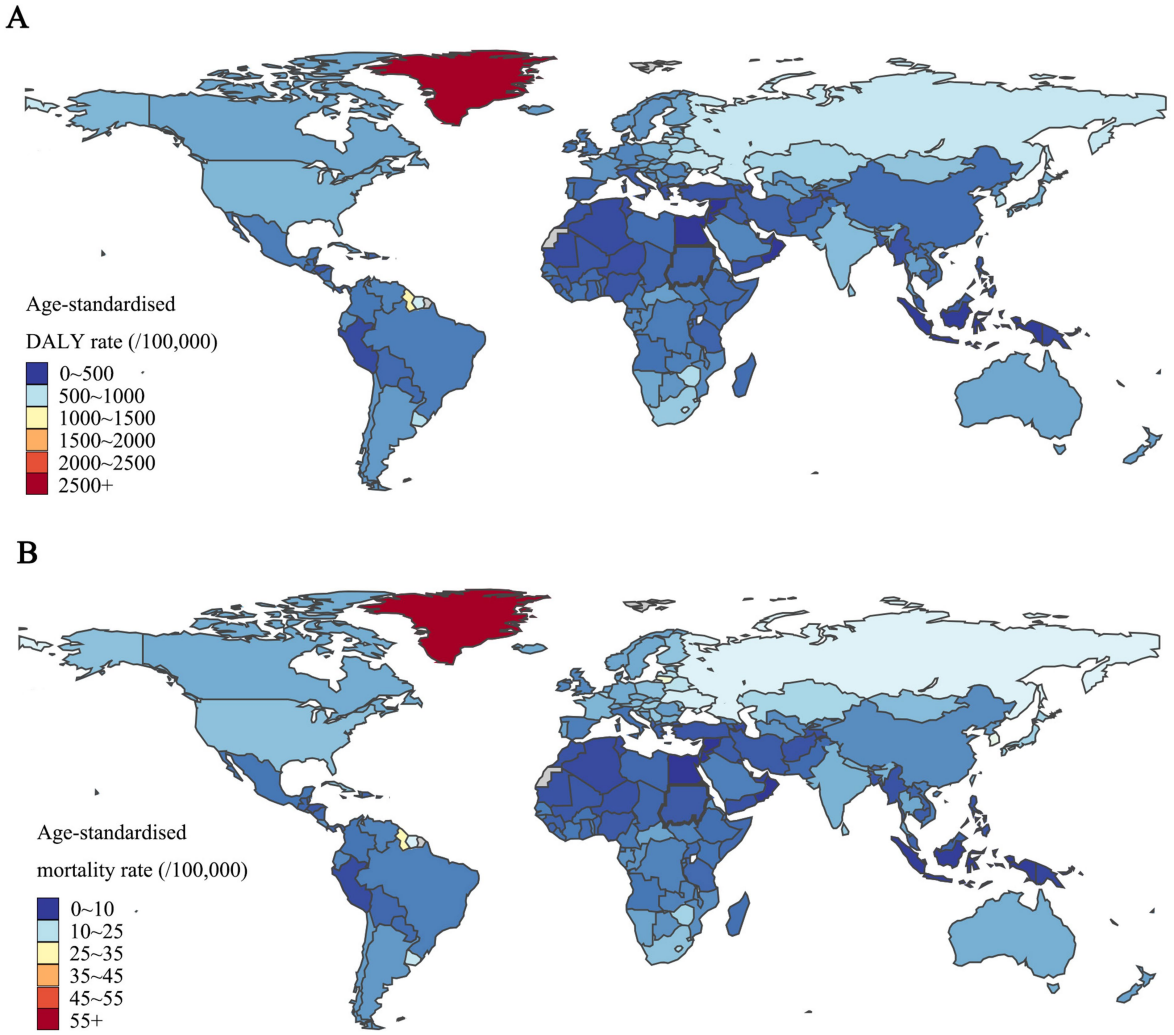
The distribution of age-standardized DALYs and mortality rates for self-harm in 204 countries and territories in 2021. **(A)** Regional distribution of the age-standardized DALYs rates. **(B)** Regional distribution of the age-standardized mortality rate. Source: GBD 2021 Visualization Tool (IHME).

In 2021, the global DALYs and death counts from self-harm were 33.5 million (95% UI: 31.3–35.8) and 746.4 thousand (95% UI: 691.8–799.8). The region with the highest number of DALYs and deaths is South Asia [11.3 million (95% UI: 9.9–12.5) for DALYs and 212.4 thousand (95%UI: 181.8–236) for deaths] in 2021. The global age-standardized DALYs and mortality rates from self-harm were 410.2/100,000 (95% UI: 383.4–438.4) and 9.0 (95% UI: 8.3–9.6). The highest age-standardized rates of DALYs and mortality were in central Europe, eastern Europe, and central Asia [692.9/100,000 (95%UI: 645.5–733.9) for DALYs rates and 14.7/100,000 (95%UI: 13.8–15.5) for mortality rate]. The further regional grouping showed that Central Europe had the highest level of self-harm burden, followed by Southern Sub-Saharan Africa, and the top 3 countries with the highest rates of self-harm globally were Greenland [2687.6/100,000 (95%UI: 2112.9–3211.3) for DALYs rates and 53.5/100,000 (95%UI: 43–63.8) for mortality rate], Guyana [1,485/100,000 (95%UI: 1153.6–1880.9) for DALYs rates and 31.5/100,000 (95%UI: 24.2–40.3) for mortality rate], and Nauru [1336.8/100,000 (95%UI: 753.1–1761.1) for DALYs rates and 24.6/100,000 (95%UI: 13.7–32.6) for mortality rate]. Detailed data were described in Table 1, Figure 3, and Supplementary Table S1.

**Table 1:** Counts and age-standardized rates of DALYs and death in GBD super-region in 2021, and percentage change in age-standardized DALYs and mortality rates from 1990 to 2021.

Categories	DALYs count in 2021 (thousands)	Age-standardized DALYs rate in 2021 (per 100,000)	Percentage change in Age-standardized DALYs rate, 1990–2021 (%)	Death count in 2021 (thousands)	Age-standardized mortality rate in 2021 (per 100,000)	Percentage change in Age-standardized mortality rate, 1990–2021 (%)
Global	33.5 (31.3, 35.8)	410.2 (383.4, 438.4)	−0.4 (−0.4, −0.3)	746.4 (691.8, 799.8)	9.0 (8.3, 9.6)	−0.4 (−0.4, −0.3)
Central Europe, eastern Europe, and central Asia	3.2 (3.0, 3.3)	692.9 (645.5, 733.9)	−0.3 (−0.3, −0.2)	73.6 (68.9, 77.6)	14.7 (13.8, 15.5)	−0.3 (−0.3, −0.3)
High income	6.0 (5.7, 6.1)	510.8 (493.6, 520.9)	−0.1 (−0.2, −0.1)	148.2 (141.1, 152.1)	10.8 (10.4, 11)	−0.2 (−0.2, −0.1)
Latin America and Caribbean	2.1 (2.0, 2.2)	334.4 (315.3, 356.1)	0.1 (0.1, 0.2)	42.3 (39.8, 45.2)	6.7 (6.3, 7.2)	0.1 (0, 0.1)
North Africa and Middle East	1.1 (1.0, 1.3)	177.4 (147.6, 203.9)	−0.3 (−0.4, −0.1)	21.2 (17.4, 24.4)	3.4 (2.8, 3.9)	−0.3 (−0.4, 0)
South Asia	11.3 (9.9, 12.5)	576.1 (502.5, 635.6)	−0.4 (−0.4, −0.1)	212.4 (181.8, 236)	11.4 (9.7, 12.7)	−0.3 (−0.4, −0.1)
Southeast Asia, east Asia, and Oceania	6.3 (5.4, 7.4)	255.0 (223.0, 299.2)	−0.6 (−0.7, −0.5)	171.4 (147.5, 204.1)	6.7 (5.8, 7.9)	−0.6 (−0.7, −0.4)
Sub − Saharan Africa	3.6 (3.1, 4.2)	423.9 (367.6, 493.4)	−0.2 (−0.3, 0)	77.2 (67.0, 89.9)	11.5 (10.1, 13.2)	−0.2 (−0.3, 0)
Sex						
Female	10.51 (9.34, 11.85)	259.05 (230.27, 291.96)	−0.5 (−0.56, −0.38)	519.01 (485.18, 556.34)	5.4 (4.76, 6.04)	−0.5 (−0.56, −0.38)
Male	23.01 (21.58, 24.6)	562.63 (527.39, 601.51)	−0.32 (−0.38, −0.24)	227.37 (200.47, 255.09)	12.81 (11.99, 13.72)	−0.34 (−0.39, −0.24)
Age group^*^						
10–14 years	0.61 (0.53, 0.73)	91.25 (79.77, 109.94)	−0.48 (−0.54, −0.26)	7.78 (6.79, 9.38)	1.17 (1.02, 1.41)	−0.48 (−0.54, −0.26)
15–19 years	3.06 (2.82, 3.39)	490.66 (451.55, 542.97)	−0.41 (−0.46, −0.27)	41.92 (38.57, 46.4)	6.72 (6.18, 7.44)	−0.41 (−0.46, −0.27)
20–24 years	4.33 (3.98, 4.67)	724.36 (666.85, 782.46)	−0.35 (−0.41, −0.23)	63.24 (58.17, 68.22)	10.59 (9.74, 11.42)	−0.34 (−0.41, −0.22)
25–29 years	4.25 (3.95, 4.55)	721.99 (671.13, 774.16)	−0.34 (−0.4, −0.23)	66.65 (61.89, 71.53)	11.33 (10.52, 12.16)	−0.33 (−0.4, −0.22)
30–34 years	3.91 (3.65, 4.22)	646.78 (603.66, 697.97)	−0.37 (−0.42, −0.27)	66.27 (61.5, 71.59)	10.96 (10.17, 11.84)	−0.37 (−0.42, −0.27)
35–39 years	3.37 (3.11, 3.6)	600.25 (554.11, 642.16)	−0.41 (−0.46, −0.32)	62.04 (56.95, 66.42)	11.06 (10.15, 11.84)	−0.41 (−0.46, −0.31)
40–44 years	2.89 (2.67, 3.1)	577.27 (534.67, 619.02)	−0.39 (−0.44, −0.29)	58.46 (54.16, 62.76)	11.69 (10.83, 12.55)	−0.39 (−0.44, −0.29)
45–49 years	2.47 (2.28, 2.66)	522.2 (481.8, 561.01)	−0.41 (−0.45, −0.33)	55.44 (50.81, 59.5)	11.71 (10.73, 12.57)	−0.41 (−0.46, −0.32)
50–54 years	2.21 (2.06, 2.38)	497.82 (462.07, 534.14)	−0.46 (−0.5, −0.37)	55.68 (51.14, 59.94)	12.51 (11.49, 13.47)	−0.46 (−0.5, −0.37)
55–59 years	1.94 (1.78, 2.09)	490.01 (450.44, 528.09)	−0.42 (−0.47, −0.32)	55.46 (50.6, 59.95)	14.01 (12.79, 15.15)	−0.42 (−0.47, −0.32)
60–64 years	1.38 (1.27, 1.47)	431.76 (398.16, 460.83)	−0.42 (−0.47, −0.33)	45.53 (41.73, 48.97)	14.23 (13.04, 15.3)	−0.42 (−0.47, −0.33)
65–69 years	1.13 (1.02, 1.22)	408.16 (370.38, 443.36)	−0.42 (−0.47, −0.33)	44 (39.77, 48.19)	15.95 (14.42, 17.47)	−0.42 (−0.47, −0.33)
70–74 years	0.84 (0.76, 0.92)	410.07 (368.59, 448.43)	−0.43 (−0.48, −0.34)	40.2 (35.76, 44.12)	19.53 (17.37, 21.43)	−0.43 (−0.49, −0.35)
75–79 years	0.55 (0.48, 0.6)	415.32 (364.75, 456.02)	−0.4 (−0.45, −0.32)	32.77 (28.52, 36.15)	24.85 (21.62, 27.41)	−0.41 (−0.46, −0.32)
80–84 years	0.34 (0.3, 0.38)	393.31 (343.72, 434.52)	−0.37 (−0.42, −0.29)	26.27 (22.78, 29.13)	30 (26.01, 33.26)	−0.38 (−0.43, −0.29)
85–89 years	0.18 (0.15, 0.2)	393 (332.91, 439.97)	−0.31 (−0.36, −0.21)	17.28 (14.54, 19.39)	37.79 (31.8, 42.42)	−0.31 (−0.37, −0.21)
90–94 years	0.06 (0.05, 0.06)	311.77 (261.99, 346.96)	−0.25 (−0.3, −0.18)	6.08 (5.02, 6.79)	34 (28.06, 37.95)	−0.26 (−0.31, −0.18)
95 + years	0.01 (0.01, 0.01)	214 (168.57, 241.46)	−0.16 (−0.22, −0.11)	1.32 (1.02, 1.5)	24.21 (18.74, 27.55)	−0.16 (−0.22, −0.1)

Source: GBD 2021 data input source tool (IHEM). ^*^For the age group shown was the crude rate.

### Sex-specific disease burden of self-harm in the 7 GBD super-regions

The global age-standardized DALYs rates for self-harm in 2021 was 562.6/100,000 for males and 259.1/100,000 for females, with the burden for males higher than for females in all 7 GBD super-regions. For females, the burden of DALYs was higher in South Asia than in other regions, with 476.7/100,000. The region with the highest burden of males was Central Europe, eastern Europe, and central Asia, with 1161.2/100,000. In 2021, the global age-standardized mortality rates for self-harm for males and females, respectively, were 12.8/100,000 and 5.4/100,000. The difference in the death burden for females between the 7 super-regions was not significant, while the highest region for males was also in Central Europe, eastern Europe, and central Asia, with 25.5/100,000 (Figure 1).

### Age-specific disease burden of self-harm in the 7 GBD super-regions

Globally, DALYs rates for self-harm in 2021 peaked in the 20–24 years age group (724.4/100,000) and then continued to decline with age. Most regions followed global trends with higher burdens in young adulthood, however, DALYs trends in Southeast Asia, east Asia, Oceania, and Sub − Saharan Africa increased to a peak in old age. In contrast, the global trend in mortality in 2021 continued to rise with age, peaking at 85–89 years age group (37.8/100,000). The 7 GBD super-regions had similar mortality trends to the global level, with Southeast Asia, east Asia, Oceania, and Sub − Saharan Africa having a greater burden of death than the global level in the 85–89 age group, at 65.5/100,000 and 79.0/100,000, respectively (Figure 2).

### The disease burden of self-harm from 1990 to 2021, and forecasts to 2050

**Figure 4:**
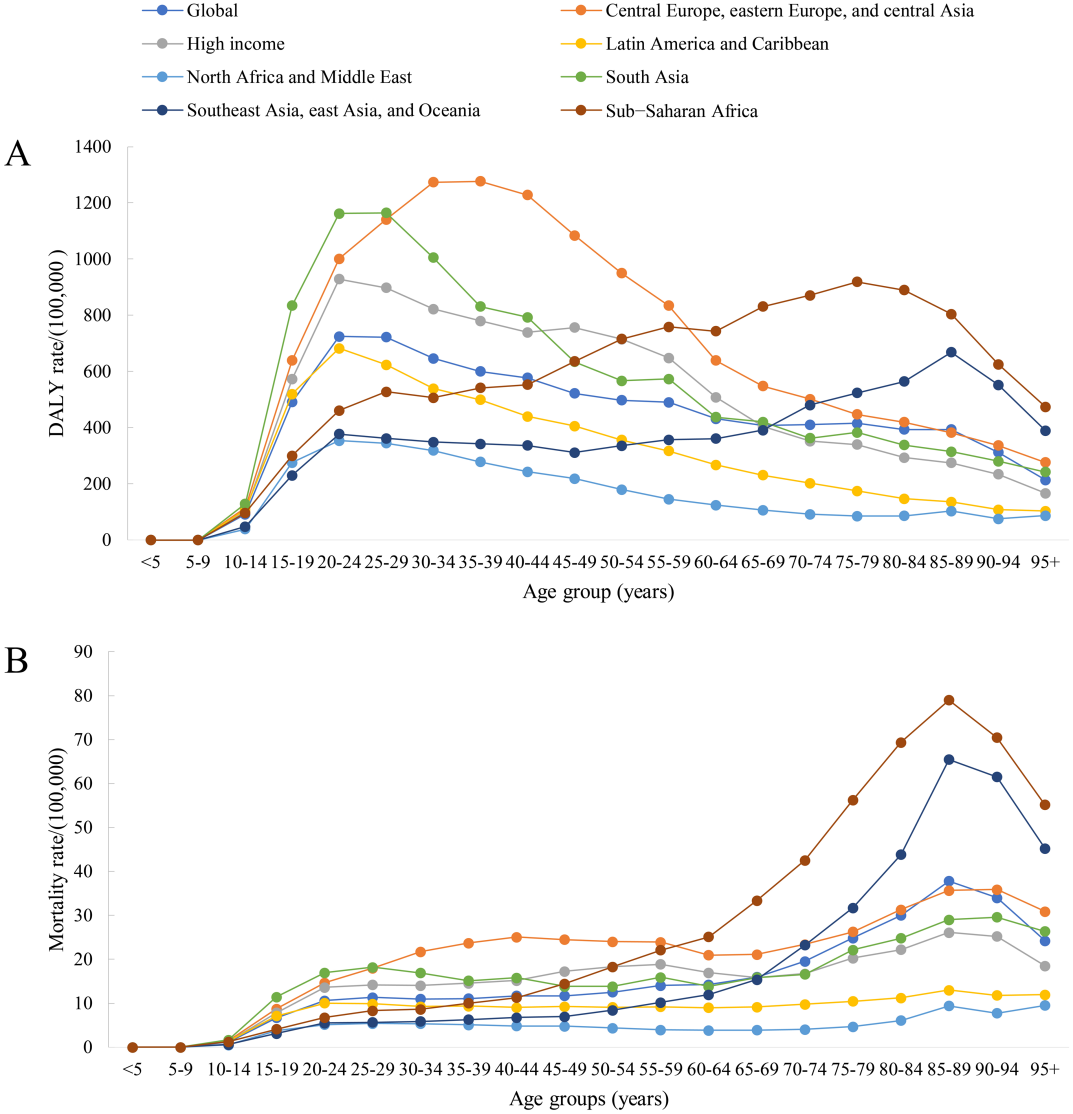
Prediction of age-standardized DALYs and mortality rates for self-harm by GBD super-region from 2022 to 2050. **(A)** Age-standardized DALYs rate for self-harm. **(B)** Age-standardized mortality rate for self-harm. Source: GBD 2021 Visualization Tool (IHME).

Globally, the percentage change in age-standardized DALYs and mortality rates both descriptively declined by 0.4% (95%CI: 0.3–0.4) over the 1990 to 2021 period. The largest observed decline in age-standardized DALYs and mortality rates from self-harm during 1990–2021 occurred in Southeast Asia, East Asia, and Oceania, with a percent change of 0.6%. Importantly, all reported differences are descriptive comparisons only; no inferential statistical analyses were conducted to evaluate these trends. And Latin America and the Caribbean was the only GBD super-region in the world where the burden of self-harm increased. The self-harm age-standardized DALYs rates for Latin America and Caribbean increased from 294.1 (95%CI: 287.5–300.9) per 100,000 in 1990 to 334.4 (95%CI: 315.3–356.1) per 100,000 in 2021, and the age-standardized mortality rate increased from 6.5 (95%CI: 6.1–6.4) per 100,000 in 1990 to 6.7 (95%CI: 6.3–7.2) per 100,000 in 2021. This rising trend underscores an urgent need for multisectoral prevention strategies—including enhanced mental healthcare access, means restriction policies, and addressing socioeconomic inequalities—to mitigate self-harm risks in the region. Although the burden of self-harm in Central Europe, eastern Europe, and central Asia had shown a fluctuating trend over 30 years, it was still much higher than in the rest of the world. In the projections for 2022 to 2050, all regions of the globe showed a decline, with Central Europe, eastern Europe, and central Asia still maintaining the highest level of self-harm burden. Detailed data were described in Figure 4 and Supplementary Tables S2–S8.

## Discussion

This study estimates the mortality and burden of self-harm at the global, regional, and national levels. It also projects the burden of disease from self-harm in selected regions in 2050. The number of self-inflicted deaths globally is estimated to be 746,388 in 2021, and we expect this to rise to approximately 877,491 by 2050, with the largest increase in sub-Saharan Africa. The global age-standardized self-inflicted mortality rate in 2021 is 9.0%, with the highest rates in the Central and Eastern Europe and Central Asia super-region, at 14.7%, and the highest rate in Greenland, at 53.5%. Self-harm has a high DALYs rates among 20-29-year-olds everywhere, but in some places, DALYs rates are also high among older people, mainly in low-SDI countries. However, it is critical to note that the results of the GBD estimates should be interpreted more cautiously for high-burden areas with wide UIs and analyzed comparatively in future studies with data from multiple sources.

While the GBD projection model accounts for demographic changes, it has inherent limitations in capturing future uncertainties. The model’s predictive power may be diminished by potential future health policy changes and the emergence of new pandemics. Long-term mortality patterns could also be influenced by shifts in the quality of health care. Additionally, projections face challenges from a wide range of environmental events, including climate change, war, and nuclear leaks. These events may lead to mass migration, food insecurity, and famine. Despite incorporating the most recent GBD data—which includes a broad array of independent factors known to affect population health—many significant potential health threats remain unaddressed in the current analysis. The inherent delay in interpreting trends in the burden of disease must also be acknowledged. Nevertheless, the GBD study remains an indispensable resource for public health. Its comprehensive scope and ability to provide standardized estimates across regions and populations offer invaluable insights that are crucial for shaping global health programs.

Self-harm could have several serious social and economic consequences globally (23, 24). For example, the average annual economic cost of suicide and non-fatal self-harm in the United States from 2015 to 2020 is approximately $510 billion, with close to 95 percent of that financial loss coming from years of life lost to suicide (25). Notably, self-harm is typically found to be concentrated among young people. During adolescence and adulthood, individuals’ self-awareness and decision-making skills are not fully developed (26). They are more susceptible to external influences that make it difficult for them to make rational decisions (27). In addition, self-harm is one of the most common reasons for emergency department visits and hospital admissions. It significantly increases the risk of future suicide deaths, meaning that more interventions are still needed. Previous research has confirmed that mental health problems may be one of the core causes of serious consequences of self-harm (2, 28).

Since 1990, we have observed a downward trend in age-standardized DALYs rates and mortality rates for self-harm. This trend can be generally observed in different regions globally (7, 29, 30). Although our data do not allow us to ascertain the reasons for the decline, it reflects a global awareness of the problem of self-harm and improvements in related interventions. Society is gradually recognizing the critical role of mental health in overall health, increasing access to mental health services and social support systems (31, 32). For instance, this study found that the burden of self-harm declined most significantly in Southeast Asia, East Asia, and Oceania. At the same time, previous studies of these regions have pursued systematic mental health initiatives during the study period, including community mental health programs (33, 34), expanded access to antidepressant therapy (35, 36), and so on. While causality needs to be further investigated, these patterns suggest that structural mental health inputs can influence the burden of self-harm from a variety of perspectives. Notably, an upward trend has been observed in Latin America and the Caribbean. This trend is mainly from central and southern Latin America, which may be related to mental stress, substance use, and crime issues (37–39). We expect the global burden of self-harm disease to decline by about 20% by 2050. The projection methodology in this study assumed that the impact of COVID-19 would decline to zero by 2030, which may underestimate the burden of self-harm due to sequelae of COVID-19 in low- and middle- income countries, where vaccination rates and postepidemic recovery are likely to be worse. And, future pandemics or long-term COVID sequelae may alter the projected burden. It is important to remember, however, that self-harm remains one of the main causes of the global burden of disease.

While this study focuses on mortality and DALY metrics, it is crucial to acknowledge that non-fatal self-harm and suicidal ideation constitute critical precursors to fatal outcomes. In the United States, for every suicide death, there are an estimated 25 non-fatal self-harm episodes, with even higher ratios in adolescent populations (40, 41). Previous studies indicate that 6 suicide rates and suicide mortality rates are much higher in self-harming patients than in the general population, underscoring the prognostic value of monitoring these behaviors (42, 43). Current surveillance systems, however, systematically underreport non-fatal self-harm due to diagnostic ambiguity (e.g., misclassification of intentional poisonings as accidents) and cultural stigma (44–46). This measurement gap is particularly acute in low-resource settings. Future burden assessments would benefit from incorporating validated instruments like the Columbia Suicide Severity Rating Scale into routine health surveillance, particularly in high-risk regions identified in our analysis.

We observed large disparities in the burden of self-harm disease across regions. For example, Greenland had the highest self-harm disease burden globally throughout the study period. Previous studies have suggested that this may be related to the problem of alcohol abuse (47, 48). In addition, several studies have found that the prevalence of self-harm is significantly higher in the indigenous population of Greenland than in the non-indigenous population (48–50). Rapid modernization and urbanization may have led to a lack of identity and a healthy mental health status among the indigenous population. Previous reviews have revealed risk and protective factors for suicide and suicidal behavior among Inuit in Greenland (51). The study notes that suicide rates in Greenland have risen dramatically since the 1970s, closely linked to colonial history, rapid modernization processes, intergenerational trauma, and grief. In addition, research on the Inuit in the Canadian region has identified the long-term negative impact of colonial history on Inuit mental health and community cohesion, while traditional Inuit lifestyles have been impacted by changes in the economy and urbanization, and men face challenges such as difficulty in finding employment and loss of cultural identity, which have combined to contribute to the rise in suicide rates (52, 53). However, that cultural stigma, limited mental health surveillance systems, and geographic isolation in indigenous communities may lead to underreporting of self-harm, potentially resulting in underestimation of its true burden in these populations. As an upper middle-income region, self-harm in Guyana is of concern. During the study period, Guyana had the second highest self-harm disease burden after Greenland. This may be related to the lack of local drug control policies, lack of guidance on mental health, and higher economic stress among young people (54, 55). In addition, a previous qualitative psychological study that interviewed 31 close relatives or friends of 20 Guyanese suicides found that the main causes of suicide included interpersonal conflict (e.g., domestic violence, marital separation, and financial disputes), trauma, and health problems (both physical and mental health) (56). Pesticide poisoning was the main way in which suicide was triggered by interpersonal conflict. Meanwhile, a review study combining 24 articles also identified pesticide poisoning as the most common form of suicide (55). The availability of toxic pesticides in rural areas, combined with poor regulation and weak healthcare infrastructure, makes impulsive self-harm even more lethal. This risk is further exacerbated among populations engaged in agribusiness occupations. Specifically, farmers, pesticide applicators, and rural laborers have routine occupational exposure to concentrated chemical agents, which are often stored in easily accessible locations such as household sheds or field stations (57). Occupational culture also plays a role: In agrarian communities, technical familiarity with pesticides may paradoxically reduce perceived lethality, while chronic stressors like crop failure debts and climate instability compound psychological distress (58–60). These findings underscore the need for occupation-specific interventions, such as centralized pesticide lockers and mandatory mental health screening during agricultural training programs. Initial trials in selected rural regions of China and Sri Lanka are evaluating whether farmer-implemented dual-lock pesticide storage systems effectively reduce intentional self-poisoning incidents and associated fatalities (61). While these recommendations are formulated based on existing intervention frameworks, their practical efficacy requires validation through targeted pilot studies and longitudinal outcome measurements.

Our study also found significant gender differences in the burden of disease for self-harm. Males had significantly higher rates of self-harm mortality and DALYs compared to females. This is consistent with the findings of previous studies (6, 62). This may be related to males’ tendency to self-harm more lethally, in addition to males being more reluctant to seek help after an injury and more likely to leave the emergency department or refuse treatment before recommendations for continuing care are made, leading to more serious medical complications (63). On the other hand, social and cultural pressures may exacerbate males’ mental health problems, such as expectations of success and societal roles, as well as socialized restrictions on expressing emotions and seeking help (64, 65). These may lead to a higher disease burden of self-harm in males. In addition, to address the higher burden of self-harm on men, we recommend strategies to reduce toxic masculinity and social pressure. Firstly, men’s emotional expression and help-seeking can be promoted through public awareness campaigns. In addition, strengthening mental health services tailored to men’s needs, such as dedicated counseling and support groups, can help manage stress and mental health issues. Finally, policy measures should focus on integrating mental health resources to better support men in high-pressure environments.

Global and regional trends in the burden of self-harm are the result of a combination of social, cultural, and economic factors. Issues such as socioeconomic inequality, poverty, and unemployment are important drivers of self-harm (66, 67). For example, Latin America and the Caribbean are the only regions where the age-standardized burden of self-harm has risen between 1990 and 2021, which may be associated with economic pressures, lack of mental health resources, and high crime rates. In regions such as Greenland, where the burden of self-harm is significantly higher than the global average, research suggests that it is closely linked to alcohol abuse, identity crises, and the rapid modernization and intergenerational trauma experienced by Indigenous groups (68–70).

Cultural factors likewise significantly influence the distribution and patterns of self-harming behavior. Gender differences are a prominent example, with males having a substantially higher rate of self-harm mortality globally (12.8/100,000) than females (5.4/100,000). This phenomenon may be related to men’s greater tendency to use more lethal means and to be less likely to seek help given social norms (71, 72). In addition, social stigmatization of mental health problems may further exacerbate this trend in some cultures, making many people reluctant to seek professional support for fear of social ostracism (73, 74).

Economic factors play a key role in reducing or increasing the burden of self-harm. High-income areas, despite higher rates of self-harm, have significantly lower associated mortality rates than low- and middle-income areas due to the availability of mental health services (75, 76). For example, North America and Western Europe have seen a decline in the burden of self-harm in recent years, thanks to strengthened public health policies and broader mental health support systems. In sub-Saharan Africa, on the other hand, while the global burden of self-harm is projected to decline by 20 percent between 2021 and 2050, the burden in the region is trending upwards, which is largely related to lagging socio-economic development and a significant lack of resources for mental health in the area.

While the findings of this study provide valuable insights into the global burden of self-harm and its regional disparities, translating these results into actionable public health strategies is crucial for maximizing their impact. Based on our findings, we recommend the development of targeted health interventions focused on high-risk populations and regions identified in the study. For instance, given the high burden of self-harm among young adults, particularly in regions like Central Europe and South Asia, public health campaigns could promote mental health awareness and provide accessible counseling services through schools, universities, and community centers. Additionally, policy interventions that address the broader social determinants of mental health, such as improving access to education and reducing economic disparities, could be considered. However, the successful implementation of these strategies may face barriers, such as financial constraints in low-income regions, cultural resistance to discussing mental health openly, or a lack of trained mental health professionals. Therefore, it is essential to account for the feasibility of these interventions, ensuring they are adapted to local contexts and are backed by the necessary resources. Future research should focus on evaluating the effectiveness of these strategies in diverse populations and settings, and on assessing the long-term sustainability of these interventions. By aligning evidence-based recommendations with public health priorities, these findings have the potential to significantly reduce the burden of self-harm in both local and global contexts.

The stark geographic disparities revealed in this study demand context-specific policy responses. These interventions should be implemented within the framework of the WHO’s LIVE LIFE suicide prevention strategy, which emphasizes four evidence-based approaches: limiting access to lethal means, promoting responsible media reporting, fostering socio-emotional skills in youth, and strengthening early intervention systems (77). In Central and Eastern Europe, where age-standardized mortality rates (14.7/100,000) far exceed the global average (9.0/100,000), alcohol control policies should be prioritized—modeled on Lithuania’s successful alcohol tax increase (2017), which reduced 57 deaths by suicide among 25–74 years old males (78). For Asia, where pesticide self-poisoning causes an estimated 300,000 deaths annually, replicating Sri Lanka’s phased pesticide bans (1995) through regional trade agreements could have a favourable impact on self-harm (79). In Latin America, integrating mental health first-aid training into secondary curricula and regulating digital platforms hosting pro-self-harm content could disrupt emerging risk pathways. For Indigenous communities like Greenland’s Inuit, culturally grounded interventions—such as land-based healing programs that reconnect youth with traditional seal-hunting practices—may counter identity crises exacerbated by rapid modernization. Meanwhile, the disproportionate burden of self-harm in low-SDI countries underscores systemic gaps in mental health infrastructure. WHO reports reveal that 88% of people in low-income countries lack access to basic mental health services, with fewer than 2% of health budgets allocated to psychiatric care—far below the WHO-recommended 5% (80). Addressing these challenges requires integrating mental health into primary care—as advocated by the WHO 2030 Action Plan—paired with context-specific adaptations (e.g., pesticide regulation in agrarian regions) (45).

There were also limitations to our analyses. First, ICD coding methods for recording the underlying cause of death may vary by location and time. Self-harm as a cause of death may be over- or under-coded depending on location and time, resulting in bias. In addition, our estimates and projections do not reflect the potential impact of the COVID-19 pandemic on the burden of self-harm in 2020 and 2021. These data were not available at the time of this analysis; the figures reported here are estimates of the burden of self-harm during a non-pandemic period. Data on the impact of the COVID-19 pandemic, when available, will be incorporated into our modeling, allowing us to assess the effect of COVID-19 on the burden of self-harm between 2020 and 2023, as well as on longer-term trends. Third, this study divides the globe according to the seven super-regions of the GBD study, oversimplifying the complexity of socio-demographic development, and focusing on these regions rather than individual countries may mask differences between countries. Fourth, challenges remain in obtaining data on the burden of disease for self-harm, particularly in low-income countries. This is mainly due to sparse data, difficulty collecting representative samples, and limited data-sharing policies. A key limitation arises in interpreting elevated self-harm DALYs rates among small-population nations (e.g., Greenland, Guyana), as small denominators magnify statistical volatility and potential reporting variations. Finally, the GBD data are based on model fits rather than real-world data, and although the data already cover almost all regions and countries of the globe, there are still some countries that lack complete statistics. Due to sparse and incomplete data, this introduces a potential reporting bias in estimates of the disease burden. In addition, the analysis relies on the quality of the raw GBD data, which is subject to possible biases in the data collection and coding process between countries and regions. It may be subject to different levels of data quality. Although the GBD used rigorous statistical methods to address these uncertainties, results should be interpreted cautiously. The results of the GBD do not reflect the impact on health outcomes that do not imply morbidity in specific contexts. It is necessary to recommend further research to include functional indicators that can be incorporated into health system monitoring mechanisms. Finally, while our models implied significant associations between macro-level factors (e.g., drug control policy, urbanization rate) and self-harm burden, the ecological nature of the GBD data precludes causal inference. The relationships should be interpreted as population-level correlations rather than individual-level causal pathways. Future studies employing quasi-experimental designs are needed to establish causality.

## Conclusion

In summary, this study analyzed regional, age, and sex differences in the burden of self-injury disease and projected the burden of self-injury disease to 2050. In 2021, Central Europe had the highest burden of self-harm globally, and young people and men were disproportionately affected. Although age-standardized DALYs rates and mortality rates for self-harm had been trending downward globally since 1990, the burden from Latin America is projected to see an increase, which also needs to be focused on. To mitigate these disparities, three actionable pathways could be prioritized: (1) Strengthening community mental health networks in Central Europe through workplace partnerships and male-friendly counselling access points; (2) Investing in primary prevention across Latin America by integrating emotional coping skills into secondary school curricula and monitoring emerging risk factors like online self-harm communities; (3) Enhancing cross-national data comparability through the adoption of standardized ICD-11 case definitions in national health surveys. The findings call for strengthened mental health services, targeted interventions, and improved data collection to effectively address and reduce the devastating impact of self-harm on individuals and global society.

## Data Availability

The original contributions presented in the study are included in the article/Supplementary material, further inquiries can be directed to the corresponding author.

## Footnotes

^1^ https://vizhub.healthdata.org/gbd-results/
