# Supplementary material for: Global burden and trends of self-harm from 1990 to 2021, with predictions to 2050
Source: Front Public Health. 2025 May 14;13:1571579. doi: 10.3389/fpubh.2025.1571579 (PMC12116644; doi:10.3389/fpubh.2025.1571579)
Supplement: Supplementary file 2 [file Data_Sheet_2.pdf]

Forecasting methodology

The forecasting methodology of this study is based on the GBD 2021 Forecasting Collaborators' "Burden of disease scenarios for 204 countries and territories, 2022-2050: a forecasting analysis for the Global Burden of Disease Study 2021" (DOI: 10.1016/S0140-6736(24)00685-8). For more specific methodology refer to the above literature.

For mortality

The mortality framework utilizes the methodology of the previous GBD (doi: 10.1016/S0140-6736(20)30677-2; doi: 10.1016/S0140-6736(18)31694-5). Briefly, we independently predict etiology using a three-part model that includes (1) a basic mortality model as a function of SDI, time, and cause-specific covariates; (2) a risk factor scalar that captures the combined risk factor effects of cause-specific factors; and (3) a stochastic wander that attenuates drift for unexplained residual mortality. Below is the model used:

$$\ln(m^{T}_{ilast}) = \alpha_{ilas} + \beta_{is}\mathrm{SDI}_{lt} + \theta_{ias}t + \ln(S_{ilast}) + \varepsilon_{ilast}$$

where the first three terms of the right-hand side of the equation constitute the logarithm of the underlying (risk-deleted) mortality,

$$\ln(m^{U}_{ilast})$$

S_{ilast} is the risk factor scalar, and for the ith cause and sth sex,

$$\alpha_{ilas} \sim \mathrm{N}(\beta_{\alpha,is,}\tau^{2}_{\alpha,is})$$

is a location-age-specific random intercept, β_{is} is a global fixed slope on SDI, and

$$\theta_{ilas} \sim \mathrm{N}(\beta_{\theta,is,}\tau^{2}_{\alpha,is})$$

is an age-specific random slope on the secular time trend. o forecast the residual trends not captured by risk factors, SDI, and global secular trends in the basic model, we used an autoregressive integrated moving average (ARIMA; 0,1,0) with attenuated drift (exponential decay parameter=0·1) on all-cause mortality, and for all other causes we used an ARIMA (0,1,0) without drift. This allowed the model to capture accelerating trends in all-cause mortality that would not be captured at the detailed cause level. We then cascaded the all-cause mortality envelope down to the most detailed levels in the hierarchy by scaling the cause-specific forecasts.

The predicted YLL is calculated as the product of the predicted cause-specific mortality rate and the standardized life expectancy at each age (using the GBD 2021 reference life tables).

Non-fatal burden

In order to compute future YLDs, we first forecasted incidence and prevalence for all causes. For causes considered acute, we forecasted incidence, prevalence, and mortality using a mixed-effect model of the mortality–incidence ratios (MIRs) and mortality–prevalence ratios (MPRs). We used the model:

$$\log(R_{a,s,l,y}) = \beta_0 + \beta_1 \mathrm{SDI}_{l,y} + \pi_{0:a,s,l} + \pi_{1:a,s,l} SDI_{l,y} + \varepsilon_{a,s,l,y}$$

where $R_{a,s,l,y}$ is the age-sex-location-year-specific ratio for a given cause, with the covariate $\mathrm{SDI}_{l,y}$ being the location-year-specific SDI. $\pi_{0:a,s,l}$ is the age-sex-location-specific random intercept, $\pi_{1:a,s,l}$ is the age-sex-location-specific slope on SDI, and $\varepsilon_{a,s,l,y}$ is the residual term.

Average disability weights for sequels were calculated from the GBD 2019 estimates and multiplied by the prevalence projections to calculate YLDs projections. We summed the YLDs with the YLLs to derive the DALYs.

Supplementary Table 1. Age-standardized rates of DALY and death in 204 countries or regions in 2021

Countries or Rigions	Age-standardised DALY rate in 2021 (per 100000)	Age-standardised mortality rate in 2021 (per 100000)
GBD regions		
Central Europe, eastern Europe, and central Asia		
Central Europe	452.3 (423.9, 478.6)	10.1 (9.5, 10.8)
Eastern Europe	914 (835.2, 995)	19.2 (17.5, 20.8)
Central Asia	483.1 (436.4, 526.7)	9.7 (8.8, 10.7)
High income		
Australasia	564 (535.5, 591)	11.2 (10.6, 11.7)
High-income Asia Pacific	654.6 (576.9, 674.9)	14.1 (12.2, 14.6)
High-income North America	632.4 (612, 652.9)	12.8 (12.4, 13.2)
Southern Latin America	498.1 (475, 522)	10 (9.5, 10.5)
Western Europe	332.2 (323.2, 340.5)	7.5 (7.3, 7.8)
Latin America and Caribbean		
Caribbean	359 (315.3, 410)	8.5 (7.5, 9.6)
Andean Latin America	245.4 (201.6, 285.7)	4.8 (3.9, 5.7)
Central Latin America	338.5 (307.8, 371.4)	6.5 (5.8, 7.1)
Tropical Latin America	345.9 (335.3, 356.9)	7 (6.8, 7.2)
North Africa and Middle East	177.4 (147.6, 203.9)	3.4 (2.8, 3.9)
South Asia	576.1 (502.5, 635.6)	11.4 (9.7, 12.7)
Southeast Asia, east Asia, and Oceania		
East Asia	266.1 (221.3, 327.3)	7.2 (6, 8.8)
Oceania	274.3 (233.9, 333.6)	5.4 (4.6, 6.4)
Southeast Asia	214.2 (183, 246.3)	4.7 (4, 5.4)
Sub−Saharan Africa		
Central Sub-Saharan Africa	516.4 (396.9, 704.4)	14.4 (11, 19.1)
Eastern Sub-Saharan Africa	426.9 (365.1, 512.4)	12.2 (10.6, 14.4)
Southern Sub-Saharan Africa	779.7 (677.7, 892.5)	16.1 (14, 18.3)
Western Sub-Saharan Africa	329.3 (263.1, 390.1)	9.5 (7.5, 11)
Countries		
American Samoa	454 (351, 565)	8.4 (6.5, 10.5)
Antigua and Barbuda	51.1 (45.9, 56.5)	1.2 (1.1, 1.3)
Arab Republic of Egypt	60.4 (49.4, 72.8)	1.1 (0.9, 1.4)
Argentine Republic	488.5 (457.2, 517)	9.6 (9, 10.2)
Australia	570.2 (540.3, 599.4)	11.3 (10.7, 11.9)
Barbados	153.3 (119, 195.2)	3.5 (2.7, 4.5)
Belize	245.9 (214.8, 279.6)	5.5 (4.8, 6.2)
Bermuda	108.1 (90.8, 132.8)	2.6 (2.2, 3.2)
Bolivarian Republic of Venezuela	417.7 (322.8, 530.9)	8 (6.1, 10.2)
Bosnia and Herzegovina	331.8 (248.2, 407.7)	7.7 (5.7, 9.4)
Brunei Darussalam	166.8 (137.6, 192.2)	3.7 (2.9, 4.2)

Burkina Faso	427.5 (333.2, 553.3)	12.6 (10.2, 16.3)
Canada	574.6 (544.2, 603.4)	11.1 (10.5, 11.6)
Central African Republic	732.1 (512.2, 996)	19.6 (14, 26.2)
Commonwealth of Dominica	159.1 (123.8, 198.1)	3.7 (2.9, 4.5)
Commonwealth of the Bahamas	112 (89.6, 138.9)	2.4 (1.9, 3)
Cook Islands	558.4 (436.4, 717.7)	10.8 (8.5, 13.8)
Czech Republic	405.1 (357.7, 448)	9.2 (8.1, 10.2)
Democratic People's Republic of Korea	382.8 (277.3, 604.4)	9.1 (6.6, 14.8)
Democratic Republic of Sao Tome and Principe	66.1 (46.4, 92)	1.5 (1.1, 2.1)
Democratic Republic of the Congo	515.3 (371.4, 744.8)	14.4 (10.3, 20.6)
Democratic Republic of Timor-Leste	231.3 (169, 309.4)	4.7 (3.5, 6.5)
Democratic Socialist Republic of Sri Lanka	718.6 (511.4, 965.3)	15.1 (10.3, 20.6)
Dominican Republic	244.1 (193.9, 309.5)	5.3 (4.3, 6.8)
Eastern Republic of Uruguay	873.1 (813.8, 933.7)	18.2 (17.2, 19.4)
Federal Democratic Republic of Ethiopia	382.3 (317.9, 457.2)	11.3 (9.4, 13.5)
Federal Democratic Republic of Nepal	653 (490.7, 857.5)	14.2 (10.7, 18.5)
Federal Republic of Germany	349.3 (333.9, 365.4)	8.2 (7.8, 8.6)
Federal Republic of Nigeria	276 (202, 362.1)	8.3 (5.9, 10.6)
Federal Republic of Somalia	458 (289.4, 782)	12.9 (8.4, 21.9)
Federated States of Micronesia	1089.4 (744.6, 1408.8)	20.1 (13.6, 26.2)
Federative Republic of Brazil	347.9 (337.2, 358.8)	7.1 (6.8, 7.3)
French Republic	428.1 (402, 455.8)	10.1 (9.4, 10.8)
Gabonese Republic	489.5 (347.5, 684.2)	13.7 (10, 18.4)
Georgia	336.4 (294.2, 383.2)	7.2 (6.3, 8.2)
Grand Duchy of Luxembourg	260.6 (235.1, 286.8)	6.5 (5.9, 7.2)
Greenland	2687.6 (2112.9, 3211.3)	53.5 (43, 63.8)
Grenada	179.2 (153.9, 208.1)	4.1 (3.5, 4.8)
Guam	928.9 (813.1, 1028)	16.7 (14.6, 18.6)
Hashemite Kingdom of Jordan	58.3 (47.2, 72.1)	1.1 (0.9, 1.4)
Hellenic Republic	156.4 (146.3, 167.2)	3.6 (3.4, 3.9)
Hungary	486.5 (433.8, 536.6)	11.6 (10.4, 12.8)
Independent State of Papua New Guinea	135.8 (101.6, 231.9)	2.8 (2.1, 4.3)
Independent State of Samoa	724.2 (526.6, 944.9)	13.6 (9.9, 17.5)
Ireland	322.9 (294.2, 355.8)	7 (6.4, 7.7)
Islamic Republic of Afghanistan	249.2 (186.9, 372.6)	5.3 (4, 7.4)
Islamic Republic of Iran	226.6 (202.9, 250.5)	4.1 (3.7, 4.6)
Islamic Republic of Mauritania	205.3 (136.6, 334.7)	5.9 (3.9, 9.8)
Islamic Republic of Pakistan	336.6 (230.6, 495.5)	6.4 (4.4, 9.5)
Jamaica	56 (42.7, 72.2)	1.3 (1, 1.7)
Japan	630.4 (617.5, 642.3)	13.2 (12.9, 13.5)
Kingdom of Bahrain	195 (161.2, 235.1)	3.9 (3.3, 4.8)
Kingdom of Belgium	574.2 (540.8, 605.3)	13.1 (12.3, 13.8)
Kingdom of Bhutan	252.1 (176.9, 369.7)	5.9 (4.4, 8.2)

Kingdom of Cambodia	223.9 (163.9, 299.5)	4.9 (3.6, 6.4)
Kingdom of Denmark	316.1 (298.6, 333.3)	7.8 (7.4, 8.2)
Kingdom of Eswatini	1032.4 (698.1, 1388.7)	21.9 (15.2, 29.3)
Kingdom of Lesotho	1111.5 (778, 1498.6)	24.3 (17.2, 32.9)
Kingdom of Morocco	183.3 (119.1, 265.6)	3.6 (2.3, 5)
Kingdom of Norway	471.1 (452.8, 490.4)	9.8 (9.3, 10.2)
Kingdom of Saudi Arabia	321.9 (242.4, 418.8)	6.6 (5, 8.6)
Kingdom of Spain	231 (218.3, 244.1)	5.4 (5, 5.7)
Kingdom of Sweden	468.4 (423.2, 517.5)	9.9 (9, 11)
Kingdom of Thailand	500.1 (392.7, 614.8)	10.1 (7.8, 12.5)
Kingdom of the Netherlands	388.4 (370, 405)	8.6 (8.1, 9)
Kingdom of Tonga	269.9 (202.6, 363.6)	5.2 (3.9, 6.9)
Kyrgyz Republic	482.4 (414.8, 556.6)	10 (8.5, 11.6)
Lao People's Democratic Republic	278.4 (204.1, 371.1)	5.5 (4.1, 7.4)
Lebanese Republic	47.8 (39.7, 57.6)	0.9 (0.7, 1)
Malaysia	220 (196.5, 254.1)	5.1 (4.6, 5.8)
Mongolia	756.4 (628, 905.5)	15.2 (12.5, 18.2)
Montenegro	506.9 (425.3, 610.9)	12.1 (10.1, 14.5)
New Zealand	531.3 (502.9, 559.2)	10.2 (9.7, 10.8)
North Macedonia	245.3 (191.6, 299.6)	6.1 (4.8, 7.4)
Northern Mariana Islands	777.3 (643.8, 907.6)	15.3 (12.8, 17.8)
Palestine	48.2 (39, 56.5)	0.9 (0.7, 1.1)
People's Democratic Republic of Algeria	150.5 (104.8, 190.4)	2.9 (2, 3.6)
People's Republic of Bangladesh	193.9 (150.2, 267.8)	3.7 (2.8, 5.1)
People's Republic of China	260 (213.5, 320.2)	7.1 (5.8, 8.8)
Plurinational State of Bolivia	276.4 (193.3, 374.6)	5.7 (3.9, 7.8)
Portuguese Republic	289.1 (273.5, 306.4)	7.2 (6.8, 7.7)
Principality of Andorra	255.6 (178.5, 337.3)	5.7 (3.9, 7.6)
Principality of Monaco	547.8 (401.6, 734.4)	11.7 (8.7, 15.3)
Puerto Rico	230 (190.2, 270.4)	5.2 (4.3, 6.1)
Republic of Albania	145.9 (115.6, 181.2)	3.2 (2.6, 4.1)
Republic of Angola	484.9 (363.7, 622)	13.8 (10.3, 17.3)
Republic of Armenia	196.4 (177, 218)	4.5 (4.1, 5.1)
Republic of Austria	423.9 (401.1, 444.8)	10 (9.4, 10.5)
Republic of Azerbaijan	115.5 (86.5, 153.3)	2.3 (1.8, 3.1)
Republic of Belarus	741.3 (612.9, 879.5)	16.1 (13.2, 19.4)
Republic of Benin	356.1 (252.6, 524.3)	9.8 (7.1, 14)
Republic of Botswana	481.1 (341.2, 687.9)	10.5 (7.6, 14.6)
Republic of Bulgaria	400.4 (345.3, 458.3)	9.3 (8, 10.6)
Republic of Burundi	480.3 (363.6, 728.7)	13.8 (10.6, 20.1)
Republic of Cabo Verde	634.3 (486.9, 815.8)	15.9 (12.3, 20.1)
Republic of Cameroon	458 (300.7, 652.6)	12.4 (8.3, 17.6)
Republic of Chad	415.5 (279.8, 648.9)	11.7 (7.9, 18.9)
Republic of Chile	449.5 (426.5, 473.9)	9.2 (8.8, 9.7)

Republic of Colombia	322.6 (272.4, 376.8)	6.2 (5.2, 7.2)
Republic of Costa Rica	436.3 (392.3, 478.2)	8.9 (8, 9.8)
Republic of Croatia	376.3 (328.7, 424)	9.3 (8.2, 10.4)
Republic of Cuba	423.4 (364.7, 479.3)	11 (9.5, 12.4)
Republic of Cyprus	134.9 (113.3, 158.3)	3.2 (2.7, 3.7)
Republic of Côte d'Ivoire	442.5 (312.5, 628.6)	12.5 (9.1, 17.5)
Republic of Djibouti	295.5 (180.7, 502.2)	8.6 (5.4, 14.5)
Republic of Ecuador	434 (349.1, 525.2)	8.4 (6.7, 10.3)
Republic of El Salvador	421.1 (339.1, 514.3)	8.2 (6.6, 10.1)
Republic of Equatorial Guinea	443.1 (294.8, 652.7)	12.1 (8.4, 17.3)
Republic of Estonia	499.3 (433, 553.5)	11.2 (9.8, 12.4)
Republic of Fiji	488.7 (372.3, 628.5)	9.2 (6.9, 11.8)
Republic of Finland	565.6 (533.9, 598.8)	12 (11.3, 12.8)
Republic of Ghana	318.1 (247.8, 412.4)	8.7 (6.7, 11.1)
Republic of Guatemala	287.6 (246.6, 330.3)	5.5 (4.7, 6.3)
Republic of Guinea	324.6 (213, 495.4)	8.9 (5.9, 13.4)
Republic of Guinea-Bissau	574.4 (418, 763.5)	15.1 (11.2, 19.5)
Republic of Guyana	1485 (1153.6, 1880.9)	31.5 (24.2, 40.3)
Republic of Haiti	379.4 (241, 507.4)	8.6 (5.7, 11.5)
Republic of Honduras	198.4 (138, 283.8)	4.7 (3.5, 6.3)
Republic of Iceland	479.4 (440.1, 521)	10.3 (9.4, 11.3)
Republic of India	652.9 (543.2, 728.1)	13 (10.5, 14.5)
Republic of Indonesia	77.9 (63.7, 99.1)	1.7 (1.4, 2.1)
Republic of Iraq	233.4 (178.5, 311)	4.9 (3.7, 6.5)
Republic of Italy	184.3 (177.3, 191.6)	4.3 (4.1, 4.5)
Republic of Kazakhstan	850.2 (757.9, 938)	18 (15.9, 20.1)
Republic of Kenya	390.5 (306.9, 533.3)	11.7 (9.1, 15.8)
Republic of Kiribati	993.6 (721.1, 1267.6)	17.6 (12.8, 22.6)
Republic of Korea	764.8 (498.4, 827.7)	18.1 (11.1, 19.7)
Republic of Latvia	633.7 (559.9, 700.1)	14.3 (12.7, 15.9)
Republic of Liberia	412.8 (266.2, 613.9)	11.3 (7.6, 16)
Republic of Lithuania	925.5 (823.4, 1014.2)	20.6 (18.3, 22.7)
Republic of Madagascar	399.3 (287.1, 541.1)	11 (7.9, 14.9)
Republic of Malawi	539.2 (419.2, 691.7)	14.8 (11.7, 18.5)
Republic of Maldives	96.2 (76.7, 121.1)	2 (1.6, 2.5)
Republic of Mali	290.3 (204.1, 419.2)	8.1 (5.6, 11.8)
Republic of Malta	163.1 (146.2, 178.9)	3.6 (3.2, 4)
Republic of Mauritius	457.9 (419.6, 483.6)	9.3 (8.6, 9.8)
Republic of Moldova	513.7 (462.6, 574.5)	11.3 (10.1, 12.7)
Republic of Mozambique	645.2 (453.9, 858.7)	17.7 (12.8, 22.9)
Republic of Namibia	616.7 (428, 864.7)	13.7 (9.8, 18.6)
Republic of Nauru	1336.8 (753.1, 1761.1)	24.6 (13.7, 32.6)
Republic of Nicaragua	292.4 (242.1, 349.6)	5.8 (4.8, 7)
Republic of Niue	879.7 (706.5, 1089.6)	15.4 (12.3, 19.3)

Republic of Palau	864.3 (693.4, 1097.9)	15.3 (12.4, 19.4)
Republic of Panama	253.3 (208, 301.4)	5.1 (4.2, 6.1)
Republic of Paraguay	281.8 (212.9, 354.5)	5.9 (4.5, 7.5)
Republic of Peru	142.9 (102.1, 181.6)	2.8 (2, 3.6)
Republic of Poland	579.3 (535.2, 621)	12.1 (11.2, 13.1)
Republic of Rwanda	415.9 (298.2, 599.6)	11.9 (8.6, 16.6)
Republic of San Marino	245.8 (159.6, 339.1)	5.4 (3.4, 7.6)
Republic of Senegal	428.4 (329.7, 574.2)	12.5 (9.7, 16.4)
Republic of Serbia	420.1 (351.7, 493)	10.9 (9.1, 12.8)
Republic of Seychelles	229.5 (196.6, 264.4)	5.1 (4.3, 5.8)
Republic of Sierra Leone	312.1 (201.1, 468.2)	8.5 (5.7, 12.6)
Republic of Singapore	313.1 (294.9, 333.1)	6.6 (6.2, 7)
Republic of Slovenia	434 (383.7, 486.6)	10.9 (9.6, 12.2)
Republic of South Africa	726.6 (644.5, 820.5)	14.7 (13.2, 16.7)
Republic of South Sudan	515 (371.9, 700.2)	15.1 (11.1, 20.3)
Republic of Sudan	252.6 (149, 368.7)	4.7 (2.8, 6.8)
Republic of Suriname	1045.7 (850.3, 1245.1)	22.2 (17.8, 26.9)
Republic of Tajikistan	189 (144.3, 273.1)	3.9 (2.9, 5.4)
Republic of the Congo	515.3 (385.7, 679.6)	14.2 (10.9, 18.2)
Republic of the Gambia	348.4 (229.4, 525.7)	9.6 (6.4, 14.2)
Republic of the Marshall Islands	1092.7 (668, 1446.8)	20 (12.1, 26.6)
Republic of the Niger	274.4 (174.4, 438.2)	7.9 (5.2, 12.1)
Republic of the Philippines	197.1 (159.2, 234.4)	3.8 (3.2, 4.6)
Republic of the Union of Myanmar	175.4 (122.6, 227.1)	3.6 (2.4, 4.6)
Republic of Trinidad and Tobago	552.9 (431.6, 698.8)	11.9 (9.1, 15.1)
Republic of Tunisia	148.7 (101.5, 209.8)	2.9 (1.9, 4.2)
Republic of Turkey	185.8 (137.1, 224.5)	3.6 (2.7, 4.3)
Republic of Uganda	412.1 (307.4, 519.7)	11.6 (8.8, 14.5)
Republic of Uzbekistan	489.1 (423.5, 557.3)	9.1 (7.9, 10.5)
Republic of Vanuatu	959.2 (708.8, 1225.5)	17.6 (12.8, 22.5)
Republic of Yemen	221.6 (106.4, 333.6)	4.5 (2.2, 6.5)
Republic of Zambia	541.4 (399.9, 725.1)	14.9 (11.2, 19.4)
Republic of Zimbabwe	1063.6 (688.6, 1409)	23.8 (15.3, 31.1)
Romania	351.4 (311.1, 396.2)	7.9 (7, 8.9)
Russian Federation	944.3 (870.2, 1013.3)	19.9 (18.4, 21.3)
Saint Kitts and Nevis	101.6 (82.8, 131.3)	2.4 (2, 3)
Saint Lucia	250.3 (209.1, 296.9)	5.6 (4.6, 6.7)
Saint Vincent and the Grenadines	235.9 (206.6, 272)	5.4 (4.8, 6.3)
Slovak Republic	412 (346.7, 475.6)	9.3 (7.8, 10.9)
Socialist Republic of Viet Nam	328.1 (254.7, 420.6)	7.7 (5.8, 9.7)
Solomon Islands	1076.6 (731.2, 1436.8)	19.9 (13.5, 26.8)
State of Eritrea	576.9 (409.7, 796.2)	15.7 (11.6, 21.1)
State of Israel	209.5 (195.8, 223.7)	4.8 (4.5, 5.1)
State of Kuwait	89.9 (76, 108)	1.8 (1.5, 2.1)

State of Libya	259.3 (171.4, 341.2)	4.9 (3.2, 6.8)
State of Qatar	168.3 (129, 218.3)	3.4 (2.6, 4.5)
Sultanate of Oman	52.8 (42.2, 65.2)	1 (0.8, 1.3)
Swiss Confederation	348.2 (326.2, 368.5)	8.2 (7.7, 8.6)
Syrian Arab Republic	46.4 (35.7, 61.2)	0.9 (0.7, 1.2)
Taiwan (Province of China)	479.9 (447.1, 505.4)	11.3 (10.5, 12)
Togolese Republic	460.8 (317.9, 643.3)	12.5 (8.8, 17.1)
Tokelau	921.4 (743.6, 1098.5)	15.7 (12.6, 19.1)
Turkmenistan	502 (399.6, 646.9)	9.9 (7.8, 12.8)
Tuvalu	897.6 (678, 1125.8)	16.6 (12.6, 20.8)
Ukraine	907.8 (669.1, 1185.3)	18.6 (13.5, 24.4)
Union of the Comoros	293.9 (205, 446.7)	8.3 (5.7, 12.7)
United Arab Emirates	141.9 (114, 186.6)	2.8 (2.2, 3.6)
United Kingdom of Great Britain and Northern Ireland	352 (343.9, 359)	7.2 (7.1, 7.4)
United Mexican States	347.4 (314.7, 382.4)	6.5 (5.8, 7.2)
United Republic of Tanzania	343.4 (256.1, 459.6)	9.8 (7.5, 13.1)
United States of America	638.8 (617.1, 660.3)	13 (12.6, 13.4)
United States Virgin Islands	339.8 (255.7, 461.9)	6.5 (5, 8.5)

DALY, disability-adjusted life year

Supplementary Table 2. The predicted age-standardized DALY and mortality rates (per 100,000) globally from 2022 to 2050.

Global	Age-standardised DALY rate (per 100000)	Age-standardised mortality rate (per 100000)
2022	407 (377.7, 434)	8.9 (8.3, 9.5)
2023	403.9 (374.8, 432.1)	8.9 (8.2, 9.5)
2024	400.4 (369.2, 428.3)	8.8 (8.1, 9.4)
2025	395.6 (362.8, 424.1)	8.7 (8, 9.3)
2026	393 (358.7, 423.5)	8.6 (7.9, 9.3)
2027	389.1 (354.7, 421.4)	8.6 (7.8, 9.3)
2028	386.3 (352.5, 419.9)	8.5 (7.8, 9.2)
2029	384.2 (348.8, 418.8)	8.5 (7.7, 9.2)
2030	378.5 (343.5, 414.7)	8.4 (7.6, 9.1)
2031	375.2 (338.3, 411.8)	8.3 (7.5, 9.1)
2032	373.1 (334.7, 411.3)	8.2 (7.4, 9.1)
2033	369.7 (329.7, 410.6)	8.2 (7.4, 9.1)
2034	366.1 (326.1, 408.7)	8.1 (7.3, 9)
2035	362.9 (321.1, 406)	8 (7.2, 9)
2036	360.3 (317.7, 405.2)	8 (7.1, 8.9)
2037	358.3 (314.9, 405.9)	8 (7, 8.9)
2038	355.5 (310.7, 404.8)	7.9 (6.9, 8.9)
2039	351.8 (306, 402.4)	7.8 (6.8, 8.9)
2040	348.7 (302, 400.8)	7.8 (6.7, 8.9)
2041	346.1 (298, 401)	7.7 (6.6, 8.9)
2042	343.8 (294.7, 400.1)	7.7 (6.6, 8.8)
2043	340.9 (290.6, 399.4)	7.6 (6.5, 8.8)
2044	338.3 (286.1, 400.2)	7.6 (6.4, 8.8)
2045	334.9 (282.6, 398.2)	7.5 (6.3, 8.8)
2046	333.8 (280.4, 399.4)	7.5 (6.3, 8.8)
2047	332.2 (277.8, 399.6)	7.4 (6.2, 8.8)
2048	329.4 (274.3, 399.4)	7.4 (6.2, 8.8)
2049	327.4 (271.1, 400.3)	7.4 (6.1, 8.8)
2050	324.2 (267.2, 399.3)	7.3 (6, 8.8)

DALY, disability-adjusted life year

Supplementary Table 3. The predicted age-standardized DALY and mortality rates (per 100,000) in Central Europe, Eastern Europe, and Central Asia from 2022 to 2050.

Central Europe, Eastern Europe, and Central Asia	Age-standardised DALY rate (per 100000)	Age-standardised mortality rate (per 100000)
2022	691.2 (649.7, 737)	14.7 (13.9, 15.6)
2023	683.7 (641.8, 732)	14.6 (13.6, 15.6)
2024	679.9 (633.7, 728.7)	14.5 (13.5, 15.5)
2025	675.9 (631.4, 726.6)	14.4 (13.5, 15.5)
2026	672 (625.7, 728)	14.4 (13.4, 15.5)
2027	667 (617.6, 724)	14.3 (13.3, 15.4)
2028	662.9 (612.4, 725.3)	14.2 (13.2, 15.4)
2029	658.7 (607.4, 723.3)	14.1 (13, 15.4)
2030	656.6 (601.2, 722)	14.1 (13, 15.4)
2031	650.2 (593.6, 715.2)	14 (12.7, 15.3)
2032	646.3 (584.4, 716.4)	13.9 (12.7, 15.2)
2033	642 (578.9, 717)	13.8 (12.6, 15.2)
2034	638.5 (575.5, 711.5)	13.7 (12.4, 15.1)
2035	635.3 (569.4, 710.2)	13.7 (12.4, 15.1)
2036	631.9 (563.1, 705.6)	13.6 (12.2, 15.1)
2037	627.5 (554.4, 709.5)	13.5 (12.1, 15)
2038	624.1 (548.3, 706.1)	13.4 (11.9, 15.1)
2039	619.9 (540.6, 703.2)	13.4 (11.8, 15)
2040	616.5 (532.6, 707.2)	13.3 (11.7, 14.9)
2041	613.4 (529, 706)	13.2 (11.5, 15)
2042	608.7 (522.6, 706.4)	13.1 (11.4, 14.9)
2043	604.9 (515.2, 707.8)	13 (11.3, 15)
2044	599.5 (511.1, 709.2)	12.9 (11.1, 14.9)
2045	596.1 (502.6, 704.6)	12.9 (11, 14.9)
2046	593.4 (499.1, 702.7)	12.8 (10.9, 14.8)
2047	590.6 (492.9, 705.8)	12.8 (10.8, 14.8)
2048	585.9 (487.4, 702.2)	12.7 (10.6, 14.9)
2049	580.9 (482.3, 695.5)	12.6 (10.5, 14.8)
2050	577.6 (473.2, 694.9)	12.5 (10.4, 14.8)

DALY, disability-adjusted life year

Supplementary Table 4. The predicted age-standardized DALY and mortality rates (per 100,000) in high-income region from 2022 to 2050.

High-income	Age-standardised DALY rate (per 100000)	Age-standardised mortality rate (per 100000)
2022	507.3 (489.3, 517.8)	10.7 (10.3, 11)
2023	502.6 (484.9, 514.1)	10.7 (10.2, 10.9)
2024	498.4 (480.8, 510.6)	10.6 (10.1, 10.8)
2025	494.5 (476.5, 507)	10.5 (10, 10.8)
2026	490.6 (472.6, 503.3)	10.4 (9.9, 10.7)
2027	487.5 (468.7, 501.5)	10.3 (9.9, 10.6)
2028	483.8 (464.8, 498.7)	10.3 (9.8, 10.6)
2029	480.3 (461, 495.6)	10.2 (9.7, 10.5)
2030	477.3 (458.2, 493)	10.1 (9.6, 10.5)
2031	473.9 (454.8, 490.9)	10.1 (9.6, 10.4)
2032	470.9 (450.4, 489.5)	10 (9.5, 10.4)
2033	467.6 (446.5, 486.4)	9.9 (9.4, 10.3)
2034	464.6 (442.8, 484.7)	9.9 (9.4, 10.3)
2035	461.2 (438.1, 482.4)	9.8 (9.3, 10.2)
2036	458.4 (434.4, 481.4)	9.8 (9.2, 10.2)
2037	455.5 (430.9, 478.9)	9.7 (9.2, 10.1)
2038	453.1 (427.9, 477.3)	9.6 (9.1, 10.1)
2039	449.5 (423.6, 474.4)	9.6 (9, 10.1)
2040	446.8 (420.5, 472.8)	9.5 (8.9, 10)
2041	444.2 (416.6, 471)	9.5 (8.9, 10)
2042	441.6 (413, 470)	9.4 (8.8, 10)
2043	438.9 (408.7, 468.1)	9.4 (8.8, 9.9)
2044	436.2 (405.3, 466.1)	9.3 (8.7, 9.9)
2045	434.2 (402.6, 465)	9.3 (8.6, 9.9)
2046	431.2 (399, 463)	9.2 (8.5, 9.8)
2047	429.2 (395.8, 461.9)	9.2 (8.5, 9.8)
2048	426.5 (392.4, 460)	9.1 (8.4, 9.8)
2049	424.2 (389.5, 458.4)	9.1 (8.3, 9.7)
2050	421.9 (386.7, 457.1)	9 (8.3, 9.7)

DALY, disability-adjusted life year

Supplementary Table 5. The predicted age-standardized DALY and mortality rates (per 100,000) in Latin America and Caribbean from 2022 to 2050.

Latin America and Caribbean	Age-standardised DALY rate (per 100000)	Age-standardised mortality rate (per 100000)
2022	334.6 (316.3, 354.2)	6.7 (6.3, 7.1)
2023	334.3 (315.4, 355.3)	6.7 (6.3, 7.1)
2024	334.3 (313.5, 356.8)	6.7 (6.3, 7.1)
2025	332.5 (311, 357.6)	6.7 (6.3, 7.1)
2026	331.7 (310, 359.5)	6.6 (6.2, 7.2)
2027	329.4 (306.3, 358.2)	6.6 (6.1, 7.1)
2028	329.2 (305.3, 360.4)	6.6 (6.1, 7.2)
2029	328.1 (301.6, 360.4)	6.6 (6.1, 7.2)
2030	323.5 (295.5, 356.9)	6.5 (5.9, 7.1)
2031	322.5 (293, 356.9)	6.4 (5.9, 7.1)
2032	322.5 (291, 358.7)	6.4 (5.9, 7.1)
2033	320.1 (288, 358.4)	6.4 (5.8, 7.1)
2034	317 (283.3, 356.8)	6.3 (5.7, 7.1)
2035	315.3 (281, 356.7)	6.3 (5.6, 7.1)
2036	314.8 (277.9, 359.5)	6.3 (5.6, 7.1)
2037	314 (275.8, 360.9)	6.3 (5.5, 7.1)
2038	312.3 (272.6, 360.6)	6.2 (5.5, 7.1)
2039	309.4 (269.1, 358.7)	6.2 (5.4, 7.1)
2040	307.7 (265.2, 360.9)	6.1 (5.3, 7.1)
2041	305.7 (261.9, 361.3)	6.1 (5.2, 7.1)
2042	303.6 (257.6, 360.2)	6 (5.2, 7.1)
2043	302 (254.4, 362.2)	6 (5.1, 7.1)
2044	300.8 (251.9, 362.3)	6 (5.1, 7.1)
2045	298.1 (248.3, 361.8)	5.9 (5, 7.1)
2046	297 (246.8, 361.7)	5.9 (5, 7.1)
2047	296 (244.3, 364.5)	5.9 (4.9, 7.2)
2048	294.2 (241, 364.1)	5.9 (4.9, 7.2)
2049	292.8 (238.3, 365)	5.8 (4.8, 7.1)
2050	290.3 (234, 362.3)	5.8 (4.7, 7.1)

DALY, disability-adjusted life year

Supplementary Table 6. The predicted age-standardized DALY and mortality rates (per 100,000) in North Africa and Middle East from 2022 to 2050.

North Africa and Middle East	Age-standardised DALY rate (per 100000)	Age-standardised mortality rate (per 100000)
2022	334.6 (316.3, 354.2)	6.7 (6.3, 7.1)
2023	334.3 (315.4, 355.3)	6.7 (6.3, 7.1)
2024	334.3 (313.5, 356.8)	6.7 (6.3, 7.1)
2025	332.5 (311, 357.6)	6.7 (6.3, 7.1)
2026	331.7 (310, 359.5)	6.6 (6.2, 7.2)
2027	329.4 (306.3, 358.2)	6.6 (6.1, 7.1)
2028	329.2 (305.3, 360.4)	6.6 (6.1, 7.2)
2029	328.1 (301.6, 360.4)	6.6 (6.1, 7.2)
2030	323.5 (295.5, 356.9)	6.5 (5.9, 7.1)
2031	322.5 (293, 356.9)	6.4 (5.9, 7.1)
2032	322.5 (291, 358.7)	6.4 (5.9, 7.1)
2033	320.1 (288, 358.4)	6.4 (5.8, 7.1)
2034	317 (283.3, 356.8)	6.3 (5.7, 7.1)
2035	315.3 (281, 356.7)	6.3 (5.6, 7.1)
2036	314.8 (277.9, 359.5)	6.3 (5.6, 7.1)
2037	314 (275.8, 360.9)	6.3 (5.5, 7.1)
2038	312.3 (272.6, 360.6)	6.2 (5.5, 7.1)
2039	309.4 (269.1, 358.7)	6.2 (5.4, 7.1)
2040	307.7 (265.2, 360.9)	6.1 (5.3, 7.1)
2041	305.7 (261.9, 361.3)	6.1 (5.2, 7.1)
2042	303.6 (257.6, 360.2)	6 (5.2, 7.1)
2043	302 (254.4, 362.2)	6 (5.1, 7.1)
2044	300.8 (251.9, 362.3)	6 (5.1, 7.1)
2045	298.1 (248.3, 361.8)	5.9 (5, 7.1)
2046	297 (246.8, 361.7)	5.9 (5, 7.1)
2047	296 (244.3, 364.5)	5.9 (4.9, 7.2)
2048	294.2 (241, 364.1)	5.9 (4.9, 7.2)
2049	292.8 (238.3, 365)	5.8 (4.8, 7.1)
2050	290.3 (234, 362.3)	5.8 (4.7, 7.1)

DALY, disability-adjusted life year

Supplementary Table 7. The predicted age-standardized DALY and mortality rates (per 100,000) in South Asia from 2022 to 2050.

South Asia	Age-standardised DALY rate (per 100000)	Age-standardised mortality rate (per 100000)
2022	569.6 (489.2, 627)	11.3 (9.6, 12.6)
2023	567.1 (488, 627.3)	11.3 (9.5, 12.6)
2024	560.9 (479.6, 623.1)	11.2 (9.3, 12.4)
2025	551.7 (469.8, 613.8)	11 (9.2, 12.3)
2026	548.7 (468.3, 614.5)	10.9 (9.2, 12.3)
2027	544 (463.2, 610.9)	10.9 (9.1, 12.2)
2028	540 (458.5, 611.5)	10.8 (9.1, 12.2)
2029	538.6 (454.5, 610.8)	10.8 (9, 12.3)
2030	527.4 (443.5, 602.3)	10.5 (8.8, 12.1)
2031	523.2 (437, 601.6)	10.5 (8.6, 12.1)
2032	520.4 (431.6, 601.9)	10.4 (8.5, 12.1)
2033	514.9 (426.5, 601.6)	10.3 (8.4, 12)
2034	510.1 (419.8, 601)	10.2 (8.3, 12)
2035	505.9 (415.6, 599.2)	10.1 (8.2, 12)
2036	501.4 (409.6, 601.9)	10.1 (8.1, 12)
2037	499.3 (403.3, 604.8)	10 (8, 12.1)
2038	494.8 (397.3, 601.5)	9.9 (7.9, 12)
2039	489.6 (392.8, 600.5)	9.8 (7.8, 12)
2040	484.3 (385.2, 600.6)	9.7 (7.6, 12)
2041	479.9 (376.1, 599.3)	9.6 (7.5, 12)
2042	478.2 (371.6, 599.8)	9.6 (7.5, 12)
2043	472.5 (363.3, 599.5)	9.5 (7.3, 12)
2044	469.1 (358.5, 600.6)	9.4 (7.3, 12)
2045	462.5 (352, 597.6)	9.3 (7.1, 12)
2046	461.9 (347.9, 602.3)	9.3 (7, 12)
2047	461.5 (344.9, 605.8)	9.3 (7, 12.1)
2048	456.6 (338.2, 605.8)	9.2 (6.9, 12.1)
2049	454.4 (335.5, 611.1)	9.2 (6.8, 12.1)
2050	448.2 (328.4, 608.1)	9 (6.7, 12.1)

DALY, disability-adjusted life year

Supplementary Table 8. The predicted age-standardized DALY and mortality rates (per 100,000) in Southeast Asia, East Asia, and Oceania from 2022 to 2050.

Southeast Asia, East Asia, and Oceania	Age-standardised DALY rate (per 100000)	Age-standardised mortality rate (per 100000)
2022	253.1 (221.9, 295.5)	6.6 (5.7, 7.8)
2023	250.2 (218.6, 292.4)	6.6 (5.7, 7.6)
2024	247.9 (216.7, 290.5)	6.5 (5.6, 7.6)
2025	245.6 (214.5, 287.6)	6.4 (5.5, 7.5)
2026	244 (212.8, 285.1)	6.4 (5.5, 7.5)
2027	241.1 (209.7, 280.2)	6.3 (5.4, 7.4)
2028	239.9 (208.3, 278.5)	6.3 (5.4, 7.3)
2029	238.6 (206.7, 277.2)	6.2 (5.3, 7.3)
2030	235.9 (204.4, 274.2)	6.2 (5.3, 7.2)
2031	233.8 (202.2, 272.3)	6.1 (5.2, 7.2)
2032	233 (200.9, 272.1)	6.1 (5.1, 7.2)
2033	231.7 (199.5, 271.6)	6.1 (5.1, 7.1)
2034	229.6 (196.7, 270.5)	6 (5, 7.1)
2035	227.9 (194.8, 267.9)	6 (5, 7.1)
2036	227.3 (193.4, 269.1)	5.9 (5, 7.1)
2037	226.3 (191.7, 269.6)	5.9 (5, 7)
2038	225.4 (189.7, 270.3)	5.9 (4.9, 7)
2039	223.5 (187.6, 269)	5.8 (4.9, 7)
2040	222.4 (186.2, 268.7)	5.8 (4.8, 7)
2041	221.5 (184.2, 270)	5.8 (4.8, 6.9)
2042	220.1 (181.9, 269.1)	5.7 (4.7, 6.9)
2043	219.1 (180.6, 268.2)	5.7 (4.7, 6.9)
2044	217.9 (179.5, 267.9)	5.7 (4.6, 6.9)
2045	216.7 (177.7, 267.2)	5.6 (4.6, 6.8)
2046	216 (176.7, 267.6)	5.6 (4.5, 6.9)
2047	215 (174.4, 268)	5.6 (4.5, 6.9)
2048	214.3 (173.2, 267.8)	5.6 (4.5, 6.9)
2049	213.6 (171.3, 267.6)	5.5 (4.5, 6.9)
2050	212 (168.8, 267.8)	5.5 (4.4, 6.8)

DALY, disability-adjusted life year

Supplementary Table 9. The predicted age-standardized DALY and mortality rates (per 100,000) in Sub-Saharan Africa from 2022 to 2050.

Sub-Saharan Africa	Age-standardised DALY rate (per 100000)	Age-standardised mortality rate (per 100000)
2022	422 (363.5, 495.5)	11.5 (9.9, 13.4)
2023	418.4 (361, 492.9)	11.4 (9.9, 13.3)
2024	414.5 (358.2, 487)	11.3 (9.8, 13.2)
2025	408.5 (352.5, 481.3)	11.2 (9.6, 12.9)
2026	406 (350.6, 477.1)	11.1 (9.6, 12.8)
2027	400.9 (343.9, 468.9)	11 (9.4, 12.7)
2028	398.2 (339.5, 466.5)	10.9 (9.3, 12.6)
2029	396.5 (336.7, 464.5)	10.8 (9.3, 12.5)
2030	390 (329.6, 457.2)	10.7 (9.1, 12.3)
2031	386.7 (325.4, 454.7)	10.6 (9, 12.3)
2032	385 (322.2, 454)	10.5 (8.9, 12.3)
2033	381.7 (317.8, 453.5)	10.5 (8.8, 12.2)
2034	377.2 (313, 450)	10.3 (8.7, 12.1)
2035	373.3 (308.1, 445.4)	10.2 (8.6, 12)
2036	370.8 (305.3, 442.7)	10.2 (8.5, 11.9)
2037	369 (301.9, 441.7)	10.1 (8.4, 12)
2038	365.9 (298.9, 439.5)	10 (8.3, 11.9)
2039	361.1 (294.3, 435)	9.9 (8.2, 11.7)
2040	357.9 (290.5, 432.9)	9.8 (8.1, 11.7)
2041	355.6 (288.2, 432.8)	9.8 (8, 11.7)
2042	352.7 (285.3, 429.8)	9.7 (7.9, 11.6)
2043	350.7 (284, 429.4)	9.7 (7.9, 11.6)
2044	347.7 (282.4, 428.4)	9.6 (7.8, 11.6)
2045	344.1 (279.1, 426.6)	9.5 (7.7, 11.5)
2046	344.2 (278, 428.1)	9.5 (7.7, 11.5)
2047	341.4 (274.3, 426.3)	9.4 (7.6, 11.5)
2048	338.7 (270.9, 424.4)	9.4 (7.6, 11.4)
2049	336.2 (268.1, 422.7)	9.3 (7.5, 11.4)
2050	334 (265.4, 422.6)	9.2 (7.5, 11.4)

DALY, disability-adjusted life year
